# Guttman error graphs: a visual approach to scalability analysis

**DOI:** 10.11606/s1518-8787.2026060007015

**Published:** 2026-02-09

**Authors:** Michael Eduardo Reichenheim, Claudia Leite de Moraes, João Luiz Bastos

**Affiliations:** I Universidade do Estado do Rio de Janeiro. Instituto de Medicina Social Hésio Cordeiro. Departamento de Epidemiologia. Rio de Janeiro, RJ, Brasil; II Universidade Estácio de Sá. Programa de Pós-graduação em Saúde da Família. Rio de Janeiro, RJ, Brasil; III Universidade Federal de Santa Catarina. Departamento de Saúde Pública. Florianópolis, SC, Brasil

**Keywords:** Psychometrics, Bias, Measures in Epidemiology

## Abstract

**OBJECTIVE:**

To develop an innovative graphical tool to represent Guttman errors and facilitate scalability analysis of measurement instruments in epidemiology.

**METHODS:**

Implemented in R (RStudio), the *guttemap* function was developed to fill this gap. It provides an intuitive visual representation of Guttman errors, with color gradients that facilitate the assessment of measurement instruments, revealing internal patterns of inconsistency. The rationale underlying the proposed Guttman error map is presented, along with an annotated summary of the routine for its implementation.

**RESULTS:**

Seven synthetic examples show the potential of graphical representation in identifying problem areas and how this helps to inform adjustments and develop more robust instruments.

**CONCLUSIONS:**

With *guttemap*, Guttman error analysis becomes more accessible and interpretable, contributing to the improvement of measurement instruments and the advancement of epidemiological research.

## INTRODUCTION

Measurement instruments are fundamental to good epidemiological practice, supporting research and guiding individuals screening and follow-up, and enabling population surveillance of diseases and health conditions. The reliability and validity of these instruments are essential to ensure the quality of the information obtained^1^. It should be noted, however, that the development or cross-cultural adaptation of instruments requires considerable time and effort^2,3^.

Among the various phases and stages of instrument development, one of the least explored is internal structure validation, specifically scalability^2^. This property refers to the ability to adequately classify or rank individuals based on the items that comprise a scale. In an ideal measurement tool, an item that supposedly captures a higher intensity of the underlying construct should only be positive if items of lesser intensity have already been endorsed^4^. This implies a cumulative and hierarchical relationship, in which the items allow the increasing intensity of the construct to be adequately mapped, progressing from a state of total absence to the opposite extreme^5^. Analyzing the scalability of a measurement instrument, therefore, corresponds to assessing the extent to which this logical consistency in response patterns is preserved.

When all items on a scale fully meet this hierarchical condition, we have a perfect Guttman scaling^1,6^. However, in practical situations, inconsistency will always arise. Population-based studies often show misclassification, with respondents endorsing higher-intensity items before milder ones. These incongruous situations are described as Guttman errors (GE),^1,6^ and their quantification is essential to understanding the profile and intensity of logical (in)consistencies between items of the instrument.

One of the proposals for assessing scalability has been Loevinger’s H coefficient^7^. The coefficient explicitly quantifies *e*_*ij*_, which can be understood as the GE between items *i* and *j* on a given scale. In effect, e_*ij*_ is the ratio between the observed probability of GE for the item pair in question to the expected probability of these errors under the assumption of no association (i.e., statistical independence) between the items. It is the complements of these quantities referring to each pair of items on the scale, *h*_*ij*_ = 1 – *e*_*ij*_, that are used to calculate Loevinger’s H coefficient, both for each item and for the scale as a whole.

A surprising issue is that software packages usually report Loevinger’s coefficients for the entire scale, individual items, and each pair of items, while little attention is given to the *e*_*ij*_ themselves, which appear only as intermediate values in the calculations performed. In addition, little emphasis is placed on the interrelationships between one item and the others in the instrument. Some software programs do display the *e*_*ij*_ values (e.g., loevh^9^, coefH^10^, MSP5^11^), but, as far as we could ascertain, there is no joint and comprehensive examination, which precludes a holistic and panoramic assessment of the issue.

Understanding the scalability profile of an instrument can be complex and paralyzing due to the amount of information presented, especially for scales with many items. An alternative is to create a graphical map that utilizes a color gradient to represent the degrees of scalability and item-overlap profiles. Thus, the purpose of this article is to provide a procedure that facilitates the visualization of the scalability profile of a measurement instrument. Drawing on a previous experience, in which graphical maps were manually assembled to illustrate the internal structure of a scale under development^12^, we propose a systematic approach that automates this graphical representation. Following a section on the rationale underlying GEs, we provide a function for implementing the graphical map in R/RStudio^13,14^. Subsequently, we offer some examples of the use of the GE map in instruments with different degrees of scalability. The article concludes with comments on future perspectives aimed at expanding and enhancing the analysis of the internal structure of epidemiological measurement instruments.

## METHODS

### Rationale behind the Guttman Error Map

Following the logic of traffic lights, ranging from green to red, the graphs presented as examples in this article aim to portray a gradient ranging from the total absence to complete GE. We designed the color gradient to be easy to interpret, clearly separating shades of green (satisfactory scenario, with little GEs), yellow (acceptable and borderline GEs), and red (excessive to unacceptable GEs). To this end, we draw on the interpretation of Loevinger’s H coefficient proposed in the literature^8^. This suggests that *h* values above 0.3 indicate acceptable scalability, with higher levels representing better scalability. In this regard, Mokken^8^ suggests the following cutoffs as criteria for qualifying the coefficients (*h*, *h*_*i*_, and *h*_*ij*_): poor scalability, if < 0.3; weak, if 0.3 to < 0.4; moderate, if 0.4 to < 0.5; and strong, if > 0.5.

**Figure 1:**
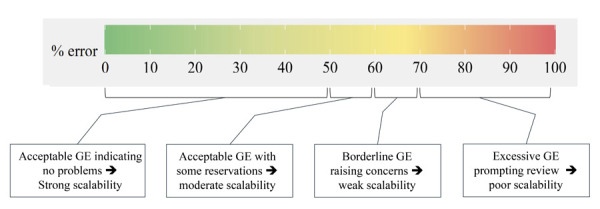
Color palette of the legend of the R function (RStudio) *guttemap* depicting levels of (un)acceptability of Guttman errors (GE).

Reiterating that *e* are the complements of *h*, that is, *h*_*ij*_ = 1 – *e*_*ij*_, and inverting Mokken’s criteria to express magnitudes in percentages, we set up the colors (graph and legend) in a gradation that depicts levels of error acceptability, *e*: ≤ 50%, suggesting no problems; > 50% to 60%, signaling admissibility of errors, albeit with reservation; > 60% to 70%, indicating that the error is borderline at a level that warrants greater attention; and > 70%, pointing to real scalability problems. This color gradient can be viewed and labeled in Figure 1, where the spectrum of full acceptability ranges from green to greenish yellow; tolerable and borderline levels appear in increasingly stronger shades of yellow; and values > 70%, which indicate problems, are colored from orange to deep red.

Another relevant aspect refers to the graphic organization of the items. For clear and consistent visualization, items need to be ordered according to their relative degrees of intensity. Items with lower endorsement frequencies would reflect higher intensities of the construct. In other words, the less frequent an item’s endorsement, the more intense the region of the construct it maps^5^. Further insights into the assumptions, interpretations, and calculations of *h* and *e* can be found in more detailed publications on the subject^4,9^. Extension to situations where items have three or more response categories is also covered in these references.

### Summary of the Routine for Implementing the Guttman Error Map

The function discussed here is called *guttemap* (Guttman Error Map) and was initially developed in a Stata ado-file^15^. From there, the routine was expanded and refined into a function in R. The improvement of the coding in R was aided by artificial intelligence^16^. The following refers exclusively to the function in R.

The R function presents a comprehensive set of arguments and functionalities, as detailed in Chart 1 ^a^. This document also provides the complete function in its appendix. To use it, the script can be copied and pasted into an ASCII text file (e.g., guttemap.R) and the code loaded into the R environment. Before doing so, the source command (“guttemap.R”) must be run in the console or by clicking the “Source” icon in the RStudio Scripts window. Eight dependent packages/libraries are required to run the function: ggplot2, ggtext, RColorBrewer, dplyr, grid, rlang, extrafont, and mokken.

**Chart 1:** Function header and initial configuration (arguments) of the R *guttemap* function*.*

Argument	Default	Instructions and explanations
data,	---	User-specified date set.
... (items)	---	Specified by the user. Items must be separated by commas, including the last one.
sw	NULL	If =TRUE, the variable containing the sample weight must be specidfied (e.g., ‘sw=data$sw_var,’. See the text for more details, as well as the *Supplementary Documentation for guttemap v 1.0*.
scl	1	Indicates the scaling of an internal variable, aiming to maximize discretion for projection to the target population if *sw* is specified. More details can be found in the text and supplementary documentation.
sclmsg	FALSE	By default, it prevents some information about the transformation and scaling of *sw* from being printed to the console. It can (should) be set to TRUE the first few times the user uses *guttemap* to guide the use of *scl*. More details can also be found in the text and supplementary documentation.
grtit	“*Guttman Error Map*”	Specifies the title of the graph. The default can be overwritten with your own title.
grfont	“*Times New Roman*”	If the user wishes, any other font allowed by the operating system can be specified.
grsave	FALSE	Does not save the graphic to the computer’s hard drive/SSD by default. If the user wishes to override this, simply indicate the name they want to give to the file to be saved.
grty	“*svg*”	Uses the SVG format by default if “grsave” is specified. This format can be converted into editable objects later. Many other formats supported by R can be specified (in quotation marks) at the user’s discretion (e.g., PDF, PostScript (PS), JPG, WMF).
titsize	18	Font size used in the title.
xlabsize	12	Font size used in horizontal labels.
ylabsize	12	Font size used in vertical labels.
valsize	3.5	Font size used for values shown within graphs, indicating the percentage of *pairwise* Guttman errors.
valformat	%.1f	It indicates the number of decimal places that values within graphs (GE) should be displayed. The default is 1 decimal place, but this can be overridden by the user (e.g., %.3f to display values to 3 decimal places).
item_position	“*bottom*”	Indicates where item labels should be positioned on the chart. The default places them below the horizontal axis to conform to tradition. However, there is an advantage to placing them at the top (specifying “top”) for vertical viewing of the GE. More details in the text.
item_order	“*decreasing*”	Specifies the order in which items are positioned on the graph. The default causes the order (from left to right and top to bottom) to automatically follow the decreasing frequency logic of the items, assuming that the less frequent the item, the more intense it is. The user can change the default by specifying “custom.” This changes the order of placement to that specified by the user in the item declaration argument.
legposit	“*bottom*”	Indicates where the legend should be positioned. In addition to the bottom, it is possible to specify “right,” ‘left,” and “top”. If the user does not want to display the legend, simply specify “none.”
legtit	“*% error*”	Specifies the caption title. The default can be overwritten with your own title.
legsize	100	Specifies the size of the legend in relation to the body of the graph. The default is maximum, but the user can decrease the size of the entire set (color gradient, title, and values).
obs	FALSE	Displays the effective sample size in the console if TRUE.
mat	FALSE	Displays the calculated matrices in the console if TRUE. Includes the Guttman and Loevinger H error matrices for pairs (H_ij_), items (H_i_), and total (H_(·)_).
tith	FALSE	If TRUE, displays the H_i_ of the items below the item labels on the horizontal axis.
itemh	FALSE	If TRUE, displays the total H_(·)_ of the scale in the chart title.
nowarn	FALSE	If TRUE, it prevents messages other than those specific to *guttemap* from being displayed.
smooth	0	Controls the level of smoothing applied to the Guttman error map. The default “smooth=0” displays a standard discrete heatmap*;* “smooth=100” creates a continuous gradient heatmap, providing a smooth color gradient effect; values between 0 and 100 apply partial smoothing, blending discrete blocks with a continuous gradient effect. More details in the text.

Most of the arguments of the *guttemap* function are self-explanatory, but a few are worth highlighting. The first refers to the decision to use the CoefH function from the mokken library. In principle, we could have programmed *guttemap* to directly perform the background calculations used to obtain e_*ij*_. However, it seemed more efficient to use this program “behind the scenes” because we also wanted to make *guttemap* viable for items with three or more response options (Likert*,* adverbial, adjectival, etc.)^1^.

Turning to CoefH revealed a limitation that had to be addressed: *guttemap* required handling data from complex samples, which is common in epidemiological research. However, the function lacked support for sample weights^10^, and ignoring them when generating graphs could yield misleading assessments of the GE. To overcome this limitation, we employed a procedural workaround that weights the data by expanding cases according to the sample-weight variable (*sw*). This strategy was adopted since the calculation of GEs depends only on point estimates and does not consider standard errors.

During the implementation of this alternative, however, we noticed using the raw values of the expansion variable could result in problems. A variable whose sample weight was, for example, between 0.311 and 2.052 would have all observations with weight < 1.0 eliminated from the expansion and, therefore, ignored in the generation of the graph. Furthermore, the expansions of the remaining observations would either not be expanded (if rounding resulted in 1.0) or, at most, would double if their sample weights were ≥ 2.0 but not exceeding 2.5. It would also be inefficient to expand already large original samples that had individuals with weights ranging, for example, from 2.699 to 5828.684.

These obstacles led us to develop a simple transformation to obtain an internal sampling weight variable that, for expansion purposes, would always start with a value ≥ 1.0 and, if necessary, still allow observations to be differentiated without losing the original metric. The equation for this transformation is:


$${\mathrm{sw}}_{1}=\mathrm{round}\left[\frac{sw_{\text{orig }}\cdot s}{sw_{mbn}}\right]$$

in which *sw*_*orig*_ is the value of the original sample weight variable to be transformed; *sw*_*min*_ is the minimum value of the original sample weight variable; *s* is the scaling factor desired by the user (requested by the scl=## argument of the function); and round() indicates rounding to the nearest integer.

Returning to the first example, the internal sampling weight corresponding to values between 0.311 and 2.052 would range from 100 to 660 when applying the above equation and setting scl=100. Regarding the second example (weights between 2.699 and 5828.684), the internal expansion variable would range from 1.0 to 2159 using the default argument scl=1. As seen, the rescaling would reduce the maximum expansion from 5828,684 to 2159. Although still computationally demanding, the procedure is clearly more efficient without any loss in the original metric.

The third comment refers the placement of item labels on the horizontal axis of the chart. Unlike the traditional practice of placing them on the bottom axis, we recommend positioning them at the top (item_position=”*TOP*”). This facilitates vertical comparison of GEs between item pairs, observing the color hue based on the distance from the “empty” diagonal graph position (as will become clearer in the graphs presented in the next section).

The fourth issue revisits what was presented in the previous section regarding the order in which the scale items are positioned in the graph. Given the rationale of linking the intensity of an item to its frequency of endorsement, there is an automated positioning from left to right. However, there are situations in which this default ordering needs to be slightly relaxed, for example, when standardizing the order of items to compare several alternative versions of a scale. The visualization of the whole set of items can be impaired if their order depends on the relative frequency in each version and does not always respect the same sequence.

A fifth point concerns the default values specified for the font size of the graph title, the labels on the horizontal and vertical axes, and the percentage values of *e*_*ij*_ at each intersection of items within the graph (Chart 1). This choice was made to better balance the layout displayed in the Plots window of RStudio. However, if the aim is to export the graph for future editing, we recommend using different specifications, such as titsize=20, xlabsize=16, ylabsize=12, and valsize=4. To change the formatting in other software (e.g., PowerPoint^®^, LibreOffice Impress, OnlyOffice, Google Slides, and Canva), we suggest exporting to SVG format (the default format in *guttemap)*, so that it can then be objectified and edited (by choosing “Convert to shape” when right-clicking with the mouse*,* positioning the cursor over the image already nested in the presentation software). It is worth noting that specifying valsize=0 (zero) allows for completely removing the Guttman error percentage values from the graph.

Finally, there is the option to smooth the image. While the unsmoothed graphic map displays well-defined color blocks, the smoothed map presents smooth transitions that gradually highlight the GE variations. In addition to an attractive aesthetic effect, it is possible to promote a representation that assumes there are continuous latent variables underlying categorical items^17,18^. The specifications from 0 to 100 of the smooth=## argument allow the user to explore a range of alternative patterns to signal the desired gradient more effectively.

## RESULTS

To illustrate the use of *guttemap*, we simulated seven simple scenarios with scales composed of five dichotomous items (i.e., those with only two response options). These simulated scales were constructed from real study data across different instruments in different sample contexts, including the use of weights and varying sample sizes. To preserve the confidentiality of the information, we adopted an emulation procedure via simulation. Although the data generated are essentially synthetic to ensure this protection, the process ensured that the overlapping profiles of the original items were preserved, allowing the intended scrutiny.

Separately for each scale, three steps were implemented to ensure informativeness. Initially, the scale was adjusted—trimmed or expanded, according to its original setup—to hold a uniform total of n = 1,000 observations. Next, for each row of the synthetic database, sampling with replacement of 1,000 records from the original database was performed. In the third step, one observation was randomly selected from the 1,000 observations to fill the current row of the synthetic database. Thus, each record (from 1 to 1,000) in this final database was formed from a sample of 1,000 candidate records (rows) from successive databases replicated from the original modified database.

**Chart 2:**
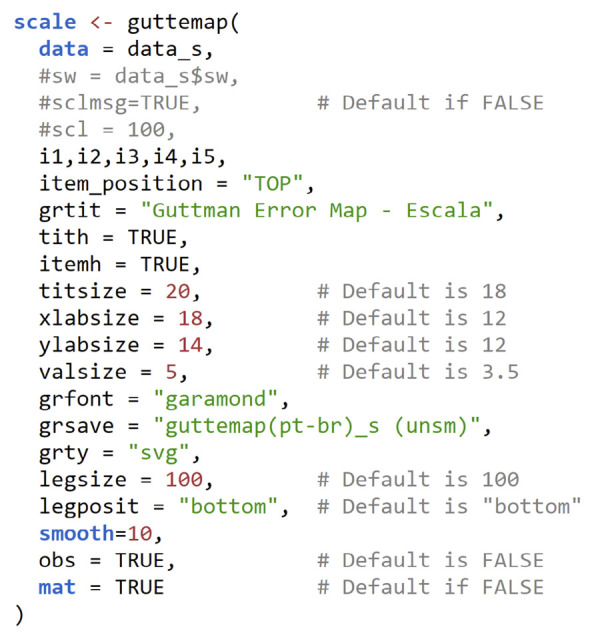
Generic and customized *guttemap* syntax (script) for generating the graphs shown in Figure 2.

**Figure 2:**
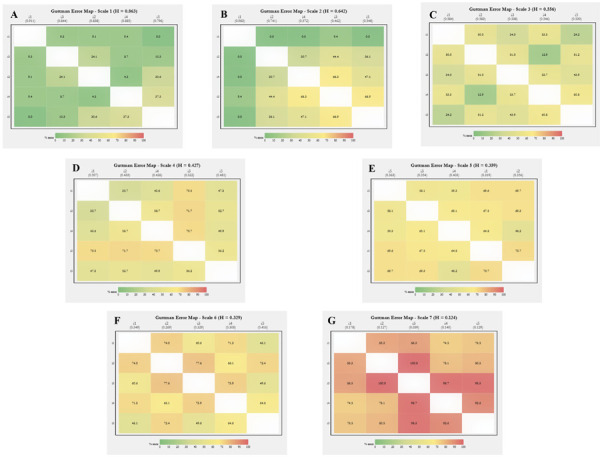
Unsmoothed graphs of the R function (RStudio) *guttemap* for seven simulated scales of decreasingly satisfactory Guttman error patterns.

The generic syntax (script) of *guttemap* shown in Chart 2 generates the graphs for the seven scales presented in Figure 2. Note that setting mat=TRUE prints the *e*_*ij*_ matrix (in percent) along with the related Loevinger coefficients, *h*, *h*_*i*_ and *h*_*ij*_ (See output in Supplementary Material S1 ^b^).

Here, three issues are worth noting. First, the graphs in Figure 2 did not use sample weights (this issue is addressed below). Second, the items are introduced from i1 to i5 in the syntax, but their positions differ in the graphs, respecting the descending order of endorsement automatically promoted by *guttemap*. Finally, the graphs were not smoothed; the smooth=10 setting shown in Chart 2 is used solely to blot out the white lines separating the boxes, which would otherwise occur if using the default (0).

Graphs A to G are ordered according to their decreasing Loevinger H coefficients, ranging from *h* = 0.863 on scale 1 to *h* = 0.124 on scale 7. The color patterns for the GEs are visibly consistent. On scale 1, for example, the hue concentrated in greens reflects the excellence of the scale, expressed in the low average error of 14.3% (or even lower, if the error between items 4 and 5 is disregarded). At the opposite extreme, orange and red tones predominate on scale 7, which would be classified as “excessive Guttman error” according to the proposal presented in Figure 1.

It is in the intermediate graphs that *guttemap* reveals its most significant potential, allowing us to visualize an overview of the internal heterogeneity of GE distribution. Even in graphs with acceptable scalability (*h*_C_ = 0.556, *h*_D_ = 0.427, and *h*_E_ = 0.359), there are marked variations, with gradations ranging from greenish to yellowish in graph C, and from yellow to light orange in graphs D and E.

Internal disparities are more evident in graphs B and F, which show very different scalability patterns. On scale 6 (F), despite an *h* slightly above the admissibility threshold, there are several confluences of items in the orange profile, showing “areas” of concern. In graph B, which, on the contrary, presents an acceptable global *h* well above the threshold of 0.50 (strong scalability), this heterogeneity is extreme. The GEs range from 0% to 66.9%, covering shades that extend from dark green to bright yellow. To emphasize, overall Loevinger’s H coefficients would have overlooked such subtleties, and thus subsequent actions aimed at reviewing and refining certain items of the instrument would not be possible.

Notably, there is the option to display the *guttemap* as a complete symmetric matrix, with the same GE values in the upper and lower triangles. This graphic layout was designed to highlight the behavior of *e*_*ij*_ vertically, focusing on how errors evolve as item intensities move away from the diagonal. In the seven graphs in Figure 2, this vertical perspective facilitates the perception of how the percentage values of GE tend to decrease as the pairs of items under analysis present successively greater differences in endorsement between each other and, therefore, how the colors of the legend (Figure 1) approach green as a result. This view is facilitated by positioning the item labels on the upper horizontal axis, whether or not they are accompanied by the respective *h*_*i*_ (itemh=TRUE).

**Chart 3:**
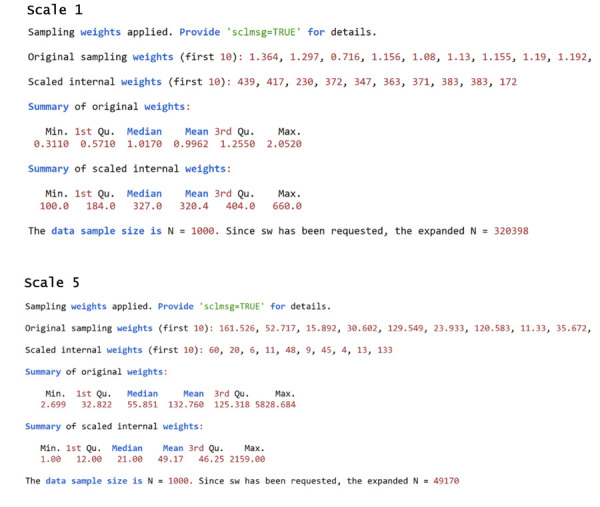
Printed content (output) in the console (R/RStudio) for scales 1 and 5 (shown in Figure 2), specifying the arguments of the *guttemap* function regarding the use of sample weights (sw=data_s$sw, scl=100, and sclmsg=TRUE).

Returning to the issue of sample weights, addressed in the section on routine synthesis, Chart 3 shows what would be printed on the console in relation to scales 1 (Figure 2A) and 5 (Figure 2E) when the three related arguments (sw=data_s$sw, scl=100, and sclmsg=TRUE) are unchecked. This information can help, early in the analysis, to decide whether it is worthwhile to adjust the factor that multiplies the sample weights. Making this adjustment can enable more precise control over these weights and enhance the sample’s representation of the population in the final analysis.

As mentioned earlier, there would be little point in expanding the weights of each individual submitted to scale 1, which range from 0.311 to 2.256, since most observations would be excluded or not even expanded. To provide context, using the original variable without any internal transformation would result in the integers 0, 1, and 2, which would lead to the exclusion of 112 observations (with a weight less than 0), the non-expansion of 777 (weight equal to 1), and only the duplication of 111 (weight between 2 and 2.5). In turn, the transformation automatically implemented by *guttemap,* but without changing the scaling (scl=1), would increase discretion by expanding seven unique values. The specification of scl=100 would result in 48 unique integer values and, of course, greater reliability in terms of the relative weighting of each observation in the sample. This exploration (R script and results) is included in Supplementary Material S2 ^c^ .

**Figure 3:**
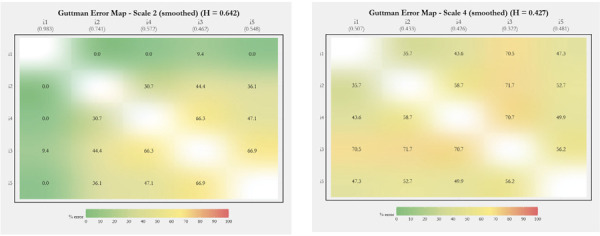
Smoothed graphs from the R (RStudio) *guttemap* function for the two scales with greater internal heterogeneity, showing a gradient transition of Guttman errors for items.

Finally, Figure 3 displays the two graphs with the greatest internal heterogeneity in the smoothed layout (smooth=80). The gradient transition of the item GEs is clearly noticeable. As in the unsmoothed graphs (Figure 2), we encourage viewing the images vertically.

## CONCLUSIONS

Measurement processes are central elements of good epidemiological practice. Without a serious commitment to the quality of the information collected, all other stages of the research process can be called into question. Although epidemiology does not rely exclusively on instruments with scalar properties, it is undeniable that dimensional scales based on the premise of scalability are widely used to measure a variety of phenomena related to health, diseases, injuries, and their associated conditions. Examples include the assessment of depression, coping*,* self-efficacy, food insecurity, intimate partner violence, resilience, self-esteem, and social support, among others. These constructs require tools that ensure consistent and valid measurements, leading to reliable interpretations that can inform public health decisions.

However, studies evaluating scalar properties are still scarce. Whether evaluating services and interventions or conducting studies on infectious diseases, chronic conditions, or psychosocial factors, a critical gap remains in the rigorous examination of these properties. This situation must be addressed beyond the usual assessments, which are limited to explorations of factorial and metric structures or external validations through correlations with other instruments or related events. Although these assessments are important within a broader procedural framework^2,19,20^, favorable evidence regarding the scalar structure of an instrument is crucial to ensure the robustness and consistency of research findings. Any unsatisfactory scaling properties that are not examined may be concealing structural weaknesses in the instruments, which often compromise their validity and, consequently, the interpretation of the study findings. Without scalability, the values of an instrument’s score are just numbers, lacking in meaning, and, as such, may not effectively represent the underlying construct^5^.

Making these psychometric analyses more accessible and interpretable is a promising way to expand their application. Here is where the present proposal comes in, offering an innovative procedure based on the identification of visual patterns (colors). The simplicity and intuitive nature of colors have the potential to “speak” more than numbers, facilitating the interpretation of results by different audiences, including researchers and health managers. In this context, *guttemap* can be a useful tool, ready for widespread use.

Although the tool is already capable of making significant contributions, improvements are clearly welcome. Among the ensuing steps, we envision: (a) the development of a complete library in R, to be made available in public repositories, expanding its accessibility; (b) the creation of a more interactive platform to facilitate both the analysis and presentation of results, thus making the tool even more user-friendly; and (c) the improvement of the core routine to implement a procedure that directly weights the observation units, eliminating the need for data expansion. Although the current approach generates unbiased results, it has limitations in terms of computational efficiency, particularly when dealing with large sample sizes or very large sample fractions, which involve high numerical values and require substantial processing power. Developing a more effective and efficient solution in this regard is a step forward in improving the use of the tool in larger-scale studies.

Finally, we encourage other researchers to join this development effort, whether to expand *guttemap* or to create analytical tools focused on health measurement instruments. Advancing this frontier strengthens epidemiology and expands its ability to respond to contemporary challenges with rigor and innovation.

## Supplementary Material

b Supplementary Material S1 is available from: https://doi.org/10.7910/DVN/8OP7ZO.

c Supplementary Material S2 is available from: https://doi.org/10.7910/DVN/8OP7ZO.

## Footnotes

a The documentation for version 1.0 of guttemap, containing details of its syntax, is available from: https://doi.org/10.7910/DVN/8OP7ZO.
