# Supplementary material for: Guttman error graphs: a visual approach to scalability analysis
Source: Rev Saude Publica. 2026 Feb 9;60:e1. doi: 10.11606/s1518-8787.2026060007015 (PMC12885155; doi:10.11606/s1518-8787.2026060007015)
Supplement: Translation

# Translation: Gráficos de erro de Guttman: uma abordagem visual para a análise de escalabilidade

**Authors:** Michael Eduardo Reichenheim, Claudia Leite de Moraes, João Luiz Bastos

**Affiliations:** I Universidade do Estado do Rio de Janeiro. Instituto de Medicina Social Hésio Cordeiro. Departamento de Epidemiologia. Rio de Janeiro, RJ, Brasil; II Universidade Estácio de Sá. Programa de Pós-graduação em Saúde da Família. Rio de Janeiro, RJ, Brasil; III Universidade Federal de Santa Catarina. Departamento de Saúde Pública. Florianópolis, SC, Brasil

**Keywords:** Psicometria, Viés, Medidas em Epidemiologia

## Abstract

**OBJETIVO:**

Desenvolver uma ferramenta gráfica inovadora para representar erros de Guttman e facilitar a análise da escalabilidade de instrumentos de aferição em epidemiologia.

**MÉTODOS:**

Implementada em R (RStudio), a função *guttemap* foi desenvolvida para preencher essa lacuna. Ela oferece uma representação visual intuitiva dos erros de Guttman, com gradientes de cores que facilitam a apreciação dos instrumentos de aferição, revelando padrões internos de inconsistência. Apresenta-se a racionalidade subjacente ao mapa de erros de Guttman proposto e uma síntese comentada da rotina para sua implementação.

**RESULTADOS:**

Com sete exemplos sintéticos, demonstra-se o potencial da representação gráfica na identificação de áreas problemáticas e como isso auxilia no embasamento de ajustes informados e o desenvolvimento de instrumentos mais robustos.

**CONCLUSÕES:**

Com o *guttemap*, a análise de erros de Guttman torna-se mais acessível e interpretável, contribuindo para o aprimoramento dos instrumentos de aferição e o avanço da pesquisa epidemiológica.

## INTRODUÇÃO

Instrumentos de mensuração constituem alicerces fundamentais da boa prática epidemiológica, sendo úteis tanto no âmbito da pesquisa quanto na identificação ou no rastreamento de indivíduos e no monitoramento populacional de doenças e agravos. A confiabilidade e a validade desses instrumentos são essenciais para garantir a qualidade da informação obtida^1^. Não deve passar despercebido, no entanto, que o desenvolvimento ou a adaptação transcultural de instrumentos exige considerável tempo e esforço^2,3^.

Entre as diversas fases e etapas de desenvolvimento de um instrumento, uma das menos exploradas é a de validação de estrutura interna, especificamente sua escalabilidade^2^. Esta se refere à capacidade de classificar ou ranquear adequadamente indivíduos com base nos itens que fazem parte de uma escala. Em uma ferramenta de mensuração ideal, um item que supostamente capta uma maior intensidade do construto subjacente somente deve ser positivado se itens de menor intensidade já o tiverem sido^4^. Isso implica uma relação cumulativa e hierárquica, na qual os itens permitem mapear adequadamente a intensidade crescente do construto, progredindo de um estado de sua total ausência até o extremo oposto^5^. Analisar a escalabilidade de um instrumento de aferição corresponde, portanto, a avaliar até que ponto essa consistência lógica nos padrões de resposta é preservada.

Quando todos os itens de uma escala atendem plenamente a essa condição hierárquica, temos um escalonamento de Guttman perfeito^1,6^. No entanto, em situações práticas, sempre haverá alguma inconsistência. Nos estudos populacionais, falhas de classificação são comuns, em que os indivíduos endossam itens de maior intensidade sem tê-los feito para aqueles de menor. Essas situações incongruentes são descritas como erros de Guttman (EG)^1,6^ e sua quantificação é essencial para entender o perfil e a intensidade de (in)consistências lógicas entre os itens do instrumento.

Uma das propostas para avaliar a escalabilidade tem sido o coeficiente H de Loevinger^7^. Para o seu cálculo, o coeficiente explicitamente quantifica *e*_*ij*_ *,* que pode ser compreendido como o EG entre os itens *i* e *j* de uma determinada escala. Com efeito, *e*_*ij*_ é a razão entre a probabilidade observada de EG para o par de itens em questão e a probabilidade esperada desses erros, assumida sob a hipótese de ausência de associação (isto é, independência estatística) entre os respectivos itens. São os complementos dessas quantidades referentes a cada par de itens da escala, *h*_*ij*_ = 1 – *e*_*ij*_, que são utilizados para o cálculo do coeficiente H de Loevinger, tanto para cada item quanto para a escala como um todo.

Uma questão surpreendente é o fato de os *softwares* apresentarem os coeficientes de Loevinger para todo o instrumento, seus itens individualmente e cada par de itens, enquanto pouca atenção é dada aos *e*_*ij*_ em si, que aparecem apenas como valores intermediários nos cálculos realizados. Além disso, pouca ênfase é conferida aos cruzamentos de um item com os demais do instrumento. Alguns *softwares* eventualmente exibem os valores *e*_*ij*_ (e.g., loevh^9^, coefH^10^, MSP5^11^), mas, até onde pudemos apurar, inexiste um exame conjunto e abrangente, o que praticamente inviabiliza uma apreciação holística e panorâmica da questão.

A apreensão do perfil de escalabilidade de um instrumento pode ser complexa e paralisante devido à quantidade de informação apresentada, principalmente em escalas com muitos itens. Uma alternativa é criar um mapa gráfico que represente os diferentes graus de escalabilidade e os perfis de imbricamento dos itens por meio de um gradiente de cores. Assim, o propósito deste artigo é fornecer um procedimento que facilite a visualização do perfil de escalabilidade de um instrumento de aferição. Com base em uma experiência prévia, na qual mapas gráficos foram elaborados manualmente para ilustrar a estrutura interna de uma escala em desenvolvimento^12^, propomos uma abordagem sistematizada que automatiza essa representação gráfica. Seguindo uma seção sobre a racionalidade subjacente aos EG, apresentamos o processo de construção de uma função para implementação do mapa gráfico em R/RStudio^13,14^. Subsequentemente, oferecemos alguns exemplos de utilização do mapa de EG em instrumentos de diferentes graus de escalabilidade. O artigo finaliza com comentários sobre perspectivas futuras voltadas à ampliação e qualificação da análise da estrutura interna de instrumentos de aferição epidemiológicos.

## MÉTODOS

### Racionalidade por trás do Mapa de Erros de Guttman

Seguindo a lógica de sinais de trânsito que vão do verde ao vermelho, os gráficos apresentados como exemplos no presente artigo procuram retratar um gradiente indo da total ausência de EG até erros completos. Projetamos o gradiente de cores para ser de fácil interpretação, nitidamente separando os tons de verde (cenário satisfatório, com pouco EG), amarelo (EG admissível e limítrofe) e vermelho (EG excessivo a inadmissível). Para isso, nos baseamos na interpretação do coeficiente H de Loevinger proposta na literatura^8^. Esta sugere que valores de *h* acima de 0,3 indicam escalabilidade aceitável, com níveis mais altos representando melhores escalabilidades. Nessa direção, Mokken^8^ sugere os seguintes cortes proporcionais como critério de qualificação dos coeficientes (*h*, *h*_*i*_ e *h*_*ij*_): escalabilidade ruim (*poor*), se < 0,3; fraca (*weak*), se 0,3 a < 0,4; moderada (*medium*), se 0,4 a < 0,5; e forte (*strong*), se > 0,5.

Reiterando que os *e* são o complemento de *h*, ou seja, *h*_*ij*_ = 1 – *e*_*ij*_, e invertendo os critérios de Mokken para expressar magnitudes em percentuais, configuramos as cores (gráfico e legenda) em uma gradação que retrata patamares de aceitabilidade dos erros, *e*: ≤ 50%, sugerindo ausência de problemas; > 50% a 60%, sinalizando admissibilidade dos erros, ainda que com reserva; > 60% a 70%, indicando que o erro é limítrofe em um patamar que suscita maior atenção; e > 70%, apontando problemas reais de escalabilidade. Esse gradiente de cores pode ser visualizado e qualificado na Figura 1, em que o espectro de plena aceitabilidade vai do verde a um amarelo esverdeado; os níveis tolerável e limítrofe aparecem em um amarelo cada vez mais forte; e os valores > 70%, que indicam problemas, são coloridos de laranja a vermelho intenso.

**Figura 1:** Paleta de cores da legenda da função R (RStudio) *guttemap* retratando patamares de (in)aceitabilidade de erros de Guttman (EG).

Outro aspecto relevante se refere à organização gráfica dos itens. Para uma visualização clara e coerente, os itens precisam ser ordenados segundo seus graus relativos de intensidade. Itens com frequências de endosso menores refletiriam intensidades mais elevadas do construto. Em outras palavras, quanto menos frequente for determinado item, mais intensa é a área do construto que ele está mapeando^5^. Detalhes sobre as premissas, interpretações e cálculos dos *h* e *e* podem ser encontrados em textos que aprofundam o conhecimento sobre o assunto^4,9^. A extensão dos cálculos para as situações de itens com três ou mais categorias de resposta também é coberta nessas referências.

### Síntese da Rotina para a Implementação do Mapa de Erros de Guttman

A função ora tratada denomina-se *guttemap* (*Guttman Error Map*) e foi desenvolvida inicialmente em um *ado-file* do Stata^15^. A partir deste, a rotina foi expandida e refinada em uma função em R. O aprimoramento da codificação em R contou com o auxílio de inteligência artificial^16^. O que se segue diz respeito exclusivamente à função em R.

A função em R apresenta um conjunto abrangente de argumentos e funcionalidades, conforme detalhado no Quadro 1 ^a^.

**Quadro 1:** Cabeçalho da função e configuração inicial (argumentos) da função R *guttemap.*

Argumento	Default	Instruções e explicações
*data,*	---	Banco de dados especificado pelo usuário.
*... (items)*	*---*	Especificado pelo usuário. Os itens devem ser seguidos de vírgula, inclusive o último.
*sw*	NULL	Caso = TRUE, a variável contendo o peso amostral deve ser indicada (e.g., ‘sw = data$sw_var,’. Veja texto para mais detalhes, assim como no *Supplementary Documentation for guttemap v 1.0*.
*scl*	1	Indica o *scaling* de uma variável interna, visando maximizar a discreção para a projeção à população-alvo se *sw* é especificado. De forma conexa, mais detalhes encontram-se no texto e documentação suplementar.
*sclmsg*	FALSE	Por padrão, impede que algumas informações sobre a transformação e *scaling* de *sw* sejam impressas no console. Pode (deve) ser configurada para TRUE nas primeiras vezes que a/o usuário usar *guttemap* para orientar o uso de *scl*. Mais detalhes encontram-se também no texto e documentação suplementar.
*grtit*	*“Guttman Error Map”*	Especifica o título do gráfico. O padrão pode ser sobrescrito com o seu próprio título.
*grfont*	*“Times New Roman”*	Caso a/o usuária/o deseje, qualquer outra fonte permitida pelo sistema operacional pode ser especificada.
*grsave*	FALSE	Não grava o gráfico no HD/SSD do computador por padrão. Caso a/o usuário deseje desconsiderar isso, basta indicar o nome que quer dar ao arquivo a gravar.
*grty*	*“svg”*	Usa o formato SVG por padrão, caso “grsave” seja indicado. Esse formato pode ser transformado em objetos editáveis subsequentemente. Muitos outros formatos suportados pelo R podem ser especificados (entre aspas) a critério da/o usuário (por exemplo, PDF, PostScript (PS), JPG, WMF).
*titsize*	18	Tamanho da fonte usada no título.
*xlabsize*	12	Tamanho da fonte usada nos rótulos horizontais
*ylabsize*	12	Tamanho da fonte usada nos rótulos verticais.
*valsize*	3.5	Tamanho da fonte usada para os valores mostrados dentro dos gráficos, indicando o percentual de *pairwise* Erros de Guttman.
*valformat*	%.1f	Indica o número de casas decimais que os valores dentro dos gráficos (EG) devem ser mostrados. O padrão é 1ª casa decimal, mas isso pede ser desconsiderado pela/o usuária/o (e.g., %.3f para mostrar os valores na 3ª casa decimal).
*item_positio*n	*“bottom”*	Indica onde os rótulos dos itens devem ser posicionados no gráfico. O padrão os coloca na parte debaixo do eixo horizontal para conformar com a tradição. No entanto, há vantagem em colocá-lo no topo (especificando “top”), visando uma apreciação vertical dos EG. Mais detalhes no texto.
*item_order*	*“decreasing”*	Especifica a ordem em que os itens são posicionados no gráfico. O padrão faz com que a ordem (da esquerda para a direita e de cima para baixo) automaticamente atenda a lógica decrescente de frequência dos itens, pressupondo que quanto menos frequente o item, mais intenso este é. A/o usuária/o pode alterar o padrão, especificando “custom”. Isso leva a ordem de posicionamento àquela especificada pela/o usuária/o no argumento de declaração dos itens.
*legposit*	*“bottom”*	Indica onde a legenda deve ser posicionada. Além da parte debaixo, é possível especificar “right”, “left” e “top”. Caso a/o usuário não queira apresentar a legenda, basta especificar “none”.
*legtit*	*“*% *error”*	Especifica o título da legenda. O padrão pode ser sobrescrito com o seu próprio título.
*legsize*	100	Especifica o tamanho da legenda em relação ao corpo do gráfico. O padrão é máximo, mas a/o usuária/o pode diminuir o tamanho de todo o conjunto (gradiente de cores, título e valores).
*obs*	FALSE	Exibe o tamanho efetivo da amostra no console se TRUE.
*mat*	FALSE	Exibe as matrizes calculadas no console se TRUE. Inclui as matrizes de erros de Guttman e dos H de Loevinger referentes aos pares (H_ij_), itens (H_i_) e total (H_(·)_).
*tith*	FALSE	Se TRUE, exibe os H_i_ dos itens abaixo do rótulo dos itens no eixo horizontal.
*itemh*	FALSE	Se TRUE, exibe o H_(·)_ total da escala no título do gráfico.
*nowarn*	FALSE	Se TRUE, evita que outras mensagens que não as específicas de *guttemap* sejam exibidas.
*smoot*h	0	Controla o nível de suavização aplicado ao mapa de erros de Guttman. O padrão “smooth = 0” exibe um *standard discrete heatmap;* “smooth = 100” cria um *heatmap* de gradiente contínuo, oferecendo um efeito de gradiente de cores suavizado; valores entre 0 e 100 aplicam suavização parcial, mesclando blocos discretos com um efeito de gradiente contínuo. Mais detalhes no texto.

Esse documento também oferece a função completa no seu apêndice. Para uso, esta pode ser copiada e colada em um arquivo de texto ASCII (e.g., guttemap.R) e o código do *script*, carregado no ambiente R. Antes, é preciso executar o comando source (“guttemap.R”) no console ou acionar o ícone “Source” na janela de Scripts do RStudio. Oito pacotes/bibliotecas dependentes são necessários para a execução da função: ggplot2, ggtext, RColorBrewer, dplyr, grid, rlang, extrafont e mokken.

A maioria dos argumentos da função *guttemap* é autoexplicativa, mas alguns comentários são necessários. O primeiro se refere à decisão pelo uso da função CoefH da biblioteca mokken. Em princípio, poderíamos ter programado o *guttemap* para executar diretamente os cálculos de fundo utilizados na obtenção dos *e*_*ij*_. No entanto, nos pareceu mais eficiente usar o referido programa “nos bastidores” porque queríamos também viabilizar o *guttemap* para situações envolvendo itens com três ou mais opções de resposta (*Likert*, adverbiais, adjetivais etc.)^1^.

Recorrer ao CoefH, porém, resultou em uma limitação que precisou ser enfrentada. Era necessário que o *guttemap* também lidasse com dados oriundos de amostragens complexas, algo comum em pesquisas epidemiológicas. Entretanto, a função não dispunha de suporte ao uso de pesos amostrais^10^ e ignorá-los na geração dos gráficos poderia levar a avaliações equivocadas dos EG. Essa limitação da função CoefH foi superada mediante um artifício processual: permitir a ponderação dos dados por meio de sua expansão com base na variável de peso amostral (*sw*). Tal estratégia foi adotada, uma vez que o cálculo dos EG depende apenas das estimativas de ponto e não considera os erros-padrão.

Durante a implementação dessa alternativa, no entanto, notamos que o uso dos valores brutos da variável de expansão poderia resultar em problemas. Uma variável cujo peso amostral fosse, por exemplo, entre 0,311 e 2,052 teria todas as observações com peso < 1,0 eliminadas da expansão e, por conseguinte, ignoradas na geração do gráfico. Ademais, as expansões das observações remanescentes ou não seriam expandidas (se o arredondamento resultasse em 1,0), ou, no máximo, dobrariam se seus pesos amostrais fossem ≥ 2,0, mas não ultrapassando 2,5. Também seria ineficiente expandir amostras originais já grandes que tivessem indivíduos com pesos abrangendo, por exemplo, 2,699 a 5828,684.

Esses entraves nos levaram a desenvolver uma transformação simples para obter uma variável de peso amostral interna que, para expansão, sempre começasse com um valor ≥ 1,0 e, se necessário, ainda permitisse diferenciar as observações sem perder a métrica original. A equação dessa transformação é:


$${\mathrm{sW}}_{i}=\mathrm{round}\left[\frac{sw_{\text{orig }}\cdot s}{sw_{\min}}\right]$$

em que *sw*_*orig*_ é o valor da variável de peso amostral original a ser transformado; *sw*_*min*_ é o valor mínimo da variável de peso amostral original; *s* é o fator de escalonamento desejado pela/o usuária/o (solicitado pelo argumento scl=## da função); e round() indica arredondamento ao número inteiro mais próximo.

Retornando ao primeiro exemplo, o peso amostral interno correspondente à variável de peso amostral entre 0,311 e 2,052 receberia valores de 100 a 660, ao se aplicar a equação acima e definir scl=100. Com relação ao segundo exemplo (pesos entre 2,699 e 5828,684), a variável de expansão interna teria amplitude de 1,0 a 2159 com o uso do argumento *default* scl=1. Como visto, a expansão máxima cairia de 5828,684 para 2159 nesse reescalonamento. Mesmo que ainda computacionalmente demandante, o procedimento é nitidamente mais eficiente sem que haja perda na métrica original.

O terceiro comentário se refere ao local onde colocar os rótulos dos itens no eixo horizontal do gráfico. Diferente da tradição de colocá-los no eixo inferior, recomendamos que sejam posicionados acima (item_position=“*TOP*”). Isso facilita a comparação vertical dos EG entre os pares de itens, observando o matiz de cores a partir do afastamento da posição gráfica diagonal ‘vazia’ (conforme ficará mais claro nos gráficos apresentados na próxima seção).

A quarta questão retoma o que foi apresentado na seção anterior sobre a ordem em que os itens da escala são posicionados no gráfico. Atendendo à racionalidade de atrelar a intensidade de um item à sua frequência de endosso, há um posicionamento automatizado da esquerda para a direita. Todavia, há situações em que essa ordenação padrão requer ser ligeiramente relaxada como, por exemplo, se há necessidade de uniformizar a ordem dos itens quando diversas versões alternativas de uma escala estão sendo comparadas. A visualização do todo pode ser prejudicada se a ordem dos itens depender da frequência relativa dos itens em cada versão e não for adaptada para que respeitem sempre a mesma sequência.

Um quinto ponto diz respeito aos valores padrão, especificados para o tamanho das fontes do título do gráfico, dos rótulos nos eixos horizontal e vertical e dos valores percentuais de *e*_*ij*_ em cada confluência de itens dentro do gráfico (Quadro 1). Essa escolha foi feita para melhor equilibrar o *layout* exibido na janela Plots do RStudio. No entanto, se a proposta é exportar o gráfico para futura edição, recomendamos usar especificações diferentes, como por exemplo, titsize=20, xlabsize=16, ylabsize=12 e valsize=4. Quando se pretende alterar a formatação em outros *softwares* (e.g., Power Point^®^, LibreOffice Impress, OnlyOffice, Google Slides e Canva), sugerimos uma exportação para o formato SVG (o formato padrão no *guttemap*), cuja imagem pode ser objetificada e detalhadamente editada em seguida (optando-se por “Converter em forma” ao se clicar no botão direito do *mouse*, posicionado o cursor sobre a imagem já aninhada no *software* de apresentação). Vale a pena observar que especificar valsize=0 (zero) permite remover completamente os valores percentuais de erros de Guttman do gráfico.

Por fim, vale comentar a opção de alisar (*smooth*) a imagem. Enquanto o mapa gráfico não alisado exibe blocos de cor bem definidos, o alisado apresenta transições suaves que evidenciam gradualmente as variações de EG. Para além de um efeito estético atraente, é possível promover uma representação que assume haver variáveis latentes contínuas por trás de itens categóricos^17,18^. As especificações de 0 a 100 do argumento smooth=## permitem que a/o usuária/o explore uma gama de padrões alternativos para melhor sinalizar o gradiente desejado.

## RESULTADOS

Para ilustrar a utilização do *guttemap*, simulamos sete cenários simples com escalas compostas por cinco itens dicotômicos (i.e., com somente duas opções de resposta). Essas escalas simuladas foram construídas a partir de dados de estudos reais que utilizaram instrumentos distintos em diferentes contextos amostrais, incluindo o uso de pesos e variados tamanhos de amostra. Para preservar a confidencialidade das informações, adotamos um procedimento de emulação via simulação. Embora os dados gerados sejam essencialmente sintéticos para garantir essa proteção, o processo assegurou que os perfis de imbricamento dos itens originais fossem preservados, permitindo o escrutínio pretendido.

Separadamente para cada escala, três passos foram implementados para garantir essa informatividade. Inicialmente, a escala foi ajustada – recortada ou ampliada, conforme sua constituição original – para conter um total uniforme de n = 1.000 observações. Em seguida, para cada linha a compor o banco sintético, foi efetuada uma amostragem com reposição de 1.000 registros da base original. No terceiro passo, dessas 1.000 observações, foi escolhida aleatoriamente uma para preencher a linha corrente do banco sintético. Assim, cada registro (de 1 a 1.000) deste banco final foi formado a partir de uma amostra entre 1.000 registros (linhas) candidatos oriundos de sucessivos bancos replicados a partir do banco modificado original.

A sintaxe (*script*) genérica do *guttemap* mostrada no Quadro 2 gera os gráficos para as sete escalas apresentados na Figura 2. Observe que a especificação de mat=TRUE aciona a impressão da matriz de *e*_*ij*_ (em porcentagens) e os três coeficientes de Loevinger relacionados, *h*, *h*_*i*_ e *h*_*ij*_ (Ver saída no Material Suplementar S1 ^b^).

**Quadro 2:** Sintaxe (*script*) genérica e customizada do *guttemap* para geração dos gráficos apresentados na Figura 2.

**Figura 2:** Gráficos não alisados da função R (RStudio) *guttemap* para sete escalas simuladas de padrões de erros de Guttman decrescentemente satisfatórios.

Aqui, três questões merecem destaque. Primeiro, que os gráficos na Figura 2 não utilizaram pesos amostrais (tal questão é tratada adiante). Segundo, que os itens são inseridos de i1 a i5 na sintaxe, mas que seus posicionamentos diferem nos gráficos, respeitando a ordem decrescente de endosso promovida automaticamente pelo *guttemap*. Por fim, que os gráficos não são alisados, com a especificação smooth=10 mostrada no Quadro 2, servindo somente para obliterar as linhas brancas de separação entre as caixas, o que ocorreria ao se usar o *default* (0).

Os gráficos A ao G estão ordenados segundo seus decrescentes coeficientes H de Loevinger, indo de *h* = 0,863 na escala 1 a *h* = 0,124 na escala 7. Os padrões de cores referentes aos EG são visivelmente coerentes. Na escala 1, por exemplo, o matiz concentrado nos verdes reflete a excelência da escala, expressa na baixa média de erros de 14,3% (ou até mais baixa, se o erro entre os itens 4 e 5 for desconsiderado). No extremo oposto, os tons laranja e vermelho predominam na escala 7, cuja qualificação seria de “erro de Guttman excessivo”, segundo a proposta apresentada na Figura 1.

Nos gráficos intermediários é que o *guttemap* revela seu maior potencial, ao permitir visualizar um panorama da heterogeneidade interna de distribuição dos EG. Mesmo nos gráficos que apresentam escalabilidades aceitáveis (*h*_C_ = 0,556, *h*_D_ = 0,427 e *h*_E_ = 0,359), observam-se variações marcantes, com gradações que vão do esverdeado ao amarelado, no gráfico C, e do amarelo ao laranja claro, nos gráficos D e E.

As disparidades internas aparecem de forma mais evidente nos gráficos B e F, que expressam padrões de escalabilidade bem diferentes. Na escala 6 (F), a despeito de um *h* ligeiramente acima do limiar de admissibilidade, há várias confluências de itens no perfil laranja, mostrando “áreas” de preocupação. No gráfico B, que, ao contrário, apresenta um *h* global confortável e bem acima do limiar de 0,50 (escalabilidade forte), essa heterogeneidade é extrema. Os EG vão de 0 a 66,9%, abrangendo tons que se estendem do verde-escuro ao amarelo forte. Para enfatizar, coeficientes globais de H de Loevinger teriam deixado passar tais sutilezas e, assim, ações subsequentes voltadas à revisão e ao refinamento de certos itens do instrumento não seriam possíveis.

Cabe aqui comentar a opção de exibir o *guttemap* como uma matriz simétrica completa, com os mesmos valores de EG nos hemitriângulos superior e inferior. Essa disposição gráfica foi projetada para destacar o comportamento dos *e*_*ij*_ verticalmente, focando em como os erros evoluem à medida que as intensidades dos itens vão se distanciando da diagonal. Nos sete gráficos da Figura 2, essa perspectiva vertical facilita a percepção de como os valores percentuais de EG tendem a diminuir conforme os pares de itens em análise apresentam diferenças de endosso sucessivamente maiores entre si e, logo, como as cores da legenda (Figura 1) se aproximam do verde em razão disso. Ajuda essa ótica posicionar os rótulos dos itens no eixo horizontal superior, sejam acompanhados ou não dos respectivos *h*_*i*_ (itemh=TRUE).

Voltando à questão do uso de pesos amostrais, abordada na seção sobre a síntese das rotinas, o Quadro 3 mostra o que se veria impresso no console em relação às escalas 1 (Figura 2A) e 5 (Figura 2E) ao se desmarcar os três argumentos conexos (sw=data_s$sw, scl=100 e sclmsg=TRUE). Essas informações podem ajudar, já no começo da análise, a decidir se vale a pena ajustar o fator que multiplica os pesos amostrais. Fazer esse ajuste pode permitir um controle mais preciso sobre esses pesos e melhorar a forma como a amostra representa a população na análise final.

**Quadro 3:** Conteúdo impresso (saída) no console (R/RStudio) relativo às escalas 1 e 5 (mostradas na Figura 2), especificando-se os argumentos da função *guttemap* quanto ao uso de pesos amostrais (sw=data_s$sw, scl=100 e sclmsg=TRUE).

Conforme mencionado anteriormente, haveria pouca utilidade em expandir os pesos de cada indivíduo submetidos à escala 1, que variam de 0,311 a 2,256, uma vez que a maioria das observações seria excluída ou sequer expandida. Para dar contexto, usar a variável original sem qualquer transformação interna resultaria nos inteiros 0, 1 e 2, o que levaria à exclusão de 112 observações (com peso inferior 0), à não expansão de 777 (peso igual a 1), e apenas à duplicação de 111 (peso entre 2 e 2,5). Por sua vez, a transformação automaticamente implementada por *guttemap*, mas sem mexer no escalonamento (scl=1), aumentaria a discreção pela expansão de 7 valores únicos. Já a especificação de scl=100 levaria a 48 valores únicos inteiros e, evidentemente, a uma fidedignidade maior quanto à ponderação relativa exercida por cada observação na amostra. Essa exploração (R *script* e resultados) consta do Material Suplementar S2 ^c^Material Suplementar S2.

Por fim, a Figura 3 mostra os dois gráficos de maior heterogeneidade interna no *layout* alisado (smooth=80). A transição em gradiente dos EG de itens é nitidamente perceptível. Como nos gráficos não alisados (Figura 2), encorajamos a apreciação das imagens na vertical.

**Figura 3:** Gráficos alisados da função R (RStudio) *guttemap* referentes às duas escalas maior heterogeneidade interna, mostrando transição em gradiente dos erros de Guttman de itens.

## CONCLUSÕES

Os processos de aferição são elementos centrais para uma boa prática epidemiológica. Sem um compromisso sério com a qualidade da informação coletada, todas as demais etapas da prática podem ser colocadas em questão. Embora a epidemiologia não dependa exclusivamente de instrumentos com propriedades escalares, é inegável que escalas dimensionais baseadas na premissa de escalabilidade são amplamente utilizadas para medir uma variedade de fenômenos em saúde, doenças, agravos e condições conexas. Exemplos incluem a avaliação de depressão, *coping*, autoeficácia, insegurança alimentar, violências entre parceiros íntimos, resiliência, autoestima e apoio social, dentre outros. Esses constructos requerem ferramentas que assegurem mensurações consistentes e válidas, levando a interpretações fidedignas que possam informar decisões em saúde pública.

No entanto, estudos que avaliem as propriedades escalares ainda são escassos. Seja para avaliar serviços e intervenções ou realizar estudos sobre doenças infecciosas, condições crônicas ou fatores psicossociais, ainda há uma lacuna crítica quanto ao exame rigoroso dessas propriedades. Essa situação deve ser enfrentada para além das avaliações usuais, limitadas a explorações de estruturas fatoriais e métricas ou validações externas por meio de correlações com outros instrumentos ou eventos relacionados. Embora essas avaliações sejam importantes dentro de uma estrutura processual mais ampla^2,19,20^, evidências favoráveis quanto à estrutura escalar de um instrumento são cruciais para garantir a solidez e a coerência dos resultados de uma pesquisa. Qualquer propriedade escalar insatisfatória que não seja examinada pode estar ocultando pontos fracos estruturais dos instrumentos que muitas vezes comprometem sua validade e, consequentemente, a interpretação dos resultados encontrados em um estudo. Sem escalabilidade, os valores de uma pontuação (escore) de um instrumento são apenas números, carentes de significado; e, como tais, podem não estar efetivamente representando o constructo subjacente^5^.

Tornar essas análises psicométricas mais acessíveis e interpretáveis é um caminho promissor para ampliar sua aplicação. É aqui que a presente proposta se insere, ao oferecer um procedimento inovador, baseado na identificação de padrões visuais (cores). A simplicidade e o caráter intuitivo proporcionados pelas cores têm o potencial de “falar” mais do que números, facilitando a interpretação de resultados por diferentes públicos, incluindo pesquisadores e gestores em saúde. Nesse contexto, o *guttemap* pode ser uma ferramenta útil, pronta para ser amplamente utilizada.

Embora já apta a oferecer contribuições significativas, aprimoramentos são claramente bem-vindos. Entre os próximos passos, vislumbramos: (a) o desenvolvimento de uma biblioteca completa em R, a ser disponibilizada em repositórios públicos, ampliando sua acessibilidade; (b) a criação de uma plataforma mais interativa para facilitar tanto a análise quanto a apresentação dos resultados e, assim, tornar a ferramenta ainda mais amigável; e (c) o aprimoramento do núcleo da rotina para implementar um procedimento que pondere diretamente as unidades de observação, dispensando a expansão de dados. Embora a atual abordagem gere resultados não enviesados, ela apresenta limitações de eficiência computacional, especialmente quando se lida com grandes tamanhos amostrais ou frações amostrais muito grandes, envolvendo valores numéricos altos e exigindo grande capacidade de processamento. Desenvolver uma solução mais efetiva e eficiente nesse sentido é um passo adiante para aprimorar o uso da ferramenta em estudos de maior escala.

Por fim, encorajamos outros pesquisadores a se unirem a este esforço de desenvolvimento, seja para expandir o *guttemap*, seja para criar ferramentas analíticas voltadas aos instrumentos de aferição em saúde. Avançar nessa fronteira é fortalecer a epidemiologia e ampliar sua capacidade de responder aos desafios contemporâneos, com rigor e inovação.

## Material Suplementar

b O Material Suplementar S1 está disponível em: https://doi.org/10.7910/DVN/8OP7ZO

c O Material Suplementar S2 está disponível em: https://doi.org/10.7910/DVN/8OP7ZO

## Footnotes

a A documentação para a versão 1.0 do guttemap, contendo os detalhes de sua sintaxe está disponível em: https://doi.org/10.7910/DVN/8OP7ZO.
